# Impact of Adding a Rapid PCR-Based Blood Culture Identification Panel to the Antimicrobial Stewardship Program of Patients with Febrile Neutropenia in a Peruvian Referral Hospital

**DOI:** 10.3390/antibiotics12040648

**Published:** 2023-03-24

**Authors:** Giancarlo Pérez-Lazo, Juana del Valle-Mendoza, Roxana Sandoval-Ahumada, Fernando Soto-Febres, Raúl Castillo-Córdova, Melissa Zárate-Tantaleán, Liliana Morales-Castillo, Celia Joanna Páucar-Miranda, Milagros Altamirano-Molina, Iván Pacheco-Modesto, Claudia Ruiz de Somocurcio-Cruzado, Denis Arana-Jurado, Carmen del Villar-Alarcón, Olga Vargas-Castro, Carol Díaz-Bardales, Bruno Guerrero-Arismendiz, Renee Eyzaguirre-Zapata, Miguel Angel Aguilar-Luis, Johanna Martins-Luna, Wilmer Silva-Caso

**Affiliations:** 1 Escuela de Medicina, Universidad César Vallejo, Piura 20001, Peru; 2 Division of Infectious Diseases, Guillermo Almenara Irigoyen National Hospital-EsSalud, Lima 15033, Peru; 3 Centro de Investigación e Innovación de la Facultad de Ciencias de la Salud, Universidad Peruana de Ciencias Aplicadas, Lima 15023, Peru; 4 Clinical Pathology Department, Guillermo Almenara Irigoyen National Hospital-EsSalud, Lima 15033, Peru; 5 Clinical Hematology Service, Guillermo Almenara Irigoyen National Hospital-EsSalud, Lima 15033, Peru

**Keywords:** febrile neutropenia, antimicrobial stewardship, Peru, outcome

## Abstract

The addition of Biofire^®^ FilmArray^®^ Blood Culture Identification panel 2 (BCID2) to the antimicrobial stewardship program (ASP) could improve outcomes in bloodstream infections (BSI) of patients with febrile neutropenia (FN). A pre- and post-quasi-experimental single-center study was conducted at a reference hospital in Peru. Three groups were considered: patients with BSI before ASP intervention (control group), patients with BSI after ASP intervention (group 1), and patients with BSI after ASP intervention plus BCID2 PCR Panel implementation (group 2). Overall, 93 patients were identified (32 control, 30 group 1, 31 group 2). The median time to effective therapy was significantly shorter in group 2 compared to group 1 and control group, respectively (3.75 vs. 10 h, *p* = 0.004; 3.75 vs. 19 h, *p* < 0.001). No significant differences in terms of relapse of bacteremia, in-hospital mortality (all cause), and 30-day-all-cause hospital readmission between the three study periods were found. The appropriateness of empirical antimicrobial use, adding or change, and the following de-escalation or discontinuation was significant when the two intervention periods were compared with the control group (*p* < 0.001). In addition to the lack of local studies documenting the microbiological profile of FN episodes, adding syndromic panels-based testing could allow for the consolidation of ASP strategies.

## 1. Introduction

Despite the improvement on preventative measures and treatment of febrile neutropenia (FN), it is still one of the most common complications in patients undergoing cancer chemotherapy, with an estimated in-hospital mortality rate of 10% [1]. Bacterial infections are common complications in patients with FN, and the epidemiology of bloodstream infection (BSI) episodes has changed in recent years [2]. Antibiotic resistance is a global threat [3], and special gram-negative bacilli have emerged as an important problem in different clinical settings, including hematological patients [4]. Moreover, the dynamics of antimicrobial resistance are influenced by local epidemiology, intensive chemotherapy, the use of invasive devices, and antimicrobial prophylaxis [5].

For instance, there is an increased rate of multidrug-resistant (MDR) gram-negative strains in countries such as Italy [6], Australia [2], China [7], Turkey [8], Egypt [9], and Colombia [10]. Mortality rates attributed to gram-negative strains and gram-positive strains are 18% and 5%, respectively [11], but they can increase to 33–71% due to carbapenem-resistant gram-negative infections [12]. Although the data from Peru remain scarce, previous studies corroborate the increased rate of MDR gram-negative strains [13].

To address this problem, the recommended strategies include developing empiric antimicrobial therapy guidelines adapted to local epidemiology [1] and performing active screening of colonizating MDR Gram-negative bacteria in rectal swabs [14], whereas failure to comply with the guidelines usually leads to greater antibiotic administration and poor clinical outcomes [15,16]. Thus, the implementation of an antimicrobial stewardship program (ASP) for patients with hematological malignancies becomes necessary, since it has shown to be an effective approach to reduce mortality [17,18,19].

The ASP has been recently implemented in some hospitals in Peru and is showing favorable results so far [20]. The aim of this program is to promote the proper use of antimicrobials to control the emergence of MDR organisms and reduce adverse effects (e.g., colitis due to *Clostridium difficile*) through strategic interventions that include prospective audit, feedback, formulary restriction and prior authorization, and prescriber’s education [21].

An important initiative of this program is diagnostic stewardship, which seeks to improve diagnostic testing, for example, through the implementation of rapid diagnostic tests for the simultaneous detection of multiple pathogens [22]. The BioFire^®^ FilmArray^®^ Blood Culture Identification 2 (BCID2) Panel is a diagnostic test that provides results for 33 pathogens and 10 antimicrobial resistance (AMR) genes from positive blood culture (PBC) specimens in about an hour. These results are vital for clinical decision regarding hospital admission, isolation, cohort, therapy, and proper use of antibiotics [23].

The aim of this study was to evaluate the impact of adding a Rapid PCR-Based Blood Culture Identification Panel to the ASP of patients with FN in a referral hospital from Lima, Peru.

## 2. Results

Overall, 93 patients with positive blood cultures who met the inclusion criteria were identified during the three study periods, with a total of 123 isolates (41 in control group, 37 in period 1, and 45 in period 2). The baseline characteristics of the patients were similar between the intervention groups and control group (before ASP implementation), except for a higher proportion of women and skin and soft tissue infections as a presumed source of bacteremia in period 2.

A higher Charlson comorbidity index was observed in group 2. A lower proportion of patients who received antibacterial prophylaxis was also observed in both intervention periods (Table 1). In control group, 11 patients received prophylaxis with ciprofloxacin and 9 with trimethoprim/sulfamethoxazole. In the intervention periods there was no prescription of quinolones as prophylaxis. During period 2, all 9/9 (100%) patients received prophylaxis with trimethoprim/sulfamethoxazole. 

**Figure 1:**
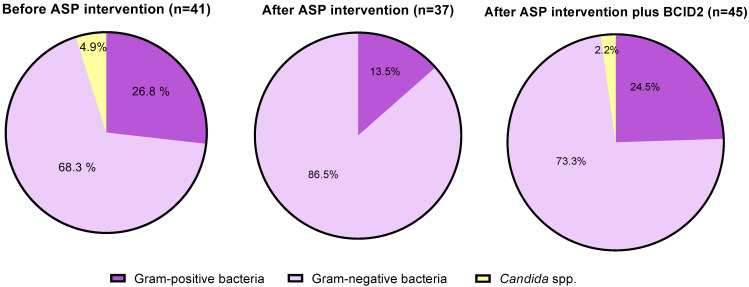
Blood culture pathogens of the included patients by groups and periods. Abbreviations: ASP = antimicrobial stewardship program, BCID2 = Blood Culture Identification 2 (BCID2) Panel.

Microbiology. BSI episodes were distributed as follows: 75.6% gram-negative, 22% gram-positive bacteria, and 2.4% for *Candida* spp. (Figure 1). No significant differences between the three study periods were found. Overall, 23 episodes of polymicrobial bacteremia were identified during the three study periods (24.7% of patients). The three most frequently isolated gram-negative species were *E. coli*, *Klebsiella pneumoniae*, and *Pseudomonas aeruginosa* during the three follow-up periods.

The median time to blood culture positivity was significantly shorter during the intervention period with BCID2 panel compared to the two previous periods (11.14 vs. 18 h; respectively) (*p* < 0.001). The median time to organism identification from the time of blood culture collection was significantly shorter in the intervention period with BCID2 panel (19.5 h) compared to the ASP implementation period (48 h) and control group (55 h) (*p* < 0.001). There were no differences in time to in vitro susceptibility results between the three study periods (Table 2).

The BCID2 panel reported 93.3% of the blood culture results in intervention period 2. Discrepancies between BCID2, culture, and phenotypic antimicrobial susceptibility test results are shown in Table 3. Three cases of discrepancy in polymicrobial cultures were detected. In patient #3, *Klebsiella pneumoniae* was identified by both methods, however the BCID2 panel additionally identified *Acinetobacter calcoaceticus-baumannii* complex, *Streptococcus* spp. and *E. coli*; this involves a change in antimicrobial therapy to colistin plus meropenem and ampicillin/sulbactam. The antimicrobial therapy in patient #11 was vancomycin + piperacillin/tazobactam, even though *Streptococcus* could not be isolated by conventional methods. Patient #31 was changed from piperacillin/tazobactam to meropenem after the detection of the CTX-M gene, however *Proteus* could not be isolated, unlike *E. coli* and *Serratia marcescens*.

In patient #4, the BCID2 panel did not detect the presence of ESBL in *Klebsiella oxytoca*, unlike what was reported by the VITEK-2 system. In patient #25, the mec A/C gene was not detected by the BCID2 panel either.

*Acinetobacter lwoffii* and *Pseudomonas putida* were identified in two cases by conventional culture, and both are not included in the BCID2 panel. In patient #27, the BCID2 panel identified *Staphylococcus epidermidis* (mec A/C gene); however, the initial preliminary examination by the microbiology laboratory indicated yeast growth, with isolation and definitive report of *Candida kefyr*. Due to this discrepancy, the patient received vancomycin and caspofungin with microbiological clearance of fungemia in control cultures at 48 h.

Regarding resistance profiles, an increase in cases of carbapenemase-producing *Enterobacteriaceae* (CPE) was observed for period 2 with the detection of the KPC gene in four isolates and OXA-48 gene in one isolate. During the two previous periods, no cases of CPE were detected. Likewise, in period 2, *Pseudomonas aeruginosa* isolates had a pan-susceptible phenotype compared to control group, where rates of MDR and extensively drug-resistant (XDR) phenotypes were 42.9% and 57.1%, respectively. After ASP intervention and BCID2, one case of *Enterococcus faecium* with vancomycin resistance (van A/van B gene) and linezolid resistance was detected (report from VITEK-2 automated system) (Table 4).

Antimicrobial utilization. The appropriateness of empirical antimicrobial use and adding or changing and the following de-escalation or discontinuation was significant when the two intervention periods were compared with the control group (*p* < 0.001) (Table 5). However, no statistically significant differences between the two intervention periods were observed. The appropriateness use of empiric therapy in intervention period 2 was 100%.

Treatment and Clinical outcomes. The median time to effective therapy was significantly shorter after ASP intervention and BCID2 compared to ASP intervention and control group, respectively (3.75 vs. 10 h, *p* = 0.004; 3.75 vs. 19 h, *p* < 0.001). No significant differences in terms of relapse of bacteremia, in-hospital mortality (all cause), 30-day-all-cause hospital readmission, and length of stay (LOS) following the first positive blood culture between the three study periods were found. However, hospital LOS was significantly shorter between ASP intervention and the BCID2 period vs. control group (28 vs. 33 days, *p* = 0.016) (Table 6). Overall mortality was 100% (n = 5) on patients who presented bacteremia due to carbapenemase-producing *Klebsiella pneumoniae*, with 3/5 (60%) presenting persistent bacteremia. Three patients with KPC-producing *Klebsiella pneumoniae* and one patient with OXA-48-producing *Klebsiella pneumoniae* bacteremia received colistin-based combination regimens.

## 3. Discussion

This study presents the first evaluation, to our knowledge, of the impact of a Rapid PCR-Based Blood Culture Identification Panel on the activities of an ASP in Peru. The study focuses on the appropriateness of antimicrobial use in different steps for a population of immunocompromised patients. In this study, we found that the addition of the BCID2 panel to ASP improves the time to effective therapy compared to the period before ASP intervention and after ASP intervention alone. These findings were similar to those reported by MacVane SH et al., where a small proportion of patients were neutropenic [24].

The clinical impact on cancer patients from rapid microbiological diagnostic tests, for example BioFire^®^ FilmArray^®^ sepsis panel (BCID) or MALDITOF (Matrix Assisted Laser Desorption Ionization—Time of Flight Mass Spectrometry), have limited data [25,26]. A quasi-experimental study reported by Buss et al. [27] analyzed the impact of BCID in treating bacteremia in cancer patients over a 4-year period. Three groups were compared, a pre-BCID period (n = 52), after BCID and before the ASP intervention (post-BCID, n = 43), and after the BCID and ASP intervention (post-ASP, n = 35). There was no significant difference in time to appropriate therapy (before BCID: 30 h; after BCID: 17 h; after ASP: 20 h; *p*= 0.43). Nevertheless, there was a difference between the pre-BCID and post-BCID groups with the multivariate regression model (pre-BCID: 38.1; post-BCID: 13.1 h; post-ASP: 8.3 h; *p* = 0.02).

Moreover, a retrospective study by Rosa et al. of 95 patients with hematologic malignancies or bone marrow transplantation showed no benefits over time for appropriate therapy using multiplex PCR blood panel identification alone or in conjunction with the ASP intervention [28]. There are limitations that may explain the non-benefit of BCID combined with ASP strategies, e.g., the general lack of understanding of these technologies among health workers. Therefore, it would be necessary to educate and explore the advantages and limitations of this approach in the onco-hematological patient population [29]. To minimize the limitation of not being familiar with this technology, we developed antibiotic treatment algorithms based on results from the BCID2 panel. To address potential uncertainties generated by reports, particularly with regard to resistance genes, we provided training and immediate feedback to both the hematology service and infection prevention and control unit.

No outcome differences on mortality at 30 days, readmission, or relapse of bacteremia were observed, even though the Charlson comorbidity index was higher in period 2. These results differ from those reported by Rosa, R.G. et al. [18], and Madran, B. et al. [19], where lower mortality rates are reported after the implementation of the ASP. Our study found a shorter hospital LOS in period 2 compared to the control group. While this finding suggests the potential for cost savings, further investigation is required to confirm the impact of ASP on this crucial metric. Specifically, longer follow-up periods and more robust data are needed to assess the impact of ASP on hospital LOS. Nevertheless, the initial reduction in LOS observed in our study provides important insight, especially for low- and middle-income countries where cost savings are critical [30].

Gram-negative bacteria were predominant as BSI agents during the three evaluated periods. This information is relevant since there are few reports of the epidemiology of BSI episodes during FN episodes in Peru. Hinojosa-Andía et al. reported a predominance of gram-negative bacteremia in two years of follow-up in another social security hospital, as ESBL-producing *Klebsiella pneumoniae* is related to mortality in patients with acute leukemia who received reinduction chemotherapy [13].

Beyond the agents that are not included in the BCID2 panel, a good performance was evident [23], especially in CPE and ESBL *Enterobacteriaceae*. In one patient with *Klebsiella oxytoca* isolate, the BCID2 panel did not identify the ESBL gene. This limitation occurs in countries with a high prevalence of ESBL genes such as Peru, whereas several types circulate (e.g., TEM, SHV) [31]. In the current era of rapid diagnostic technologies, combining different diagnostic methods to identify antimicrobial resistance mechanisms can lead to discordant results when compared to conventional microbiology laboratory data. Additionally, in the case of polymicrobial results, the use and interpretation of these tools require rationalization to enable individualized clinical decision-making. To address these challenges, it is essential to optimize the interpretation of antimicrobial susceptibility testing. Our study emphasizes the need for a case-by-case approach to the interpretation of discordant results, taking into consideration individualized clinical scenarios.

Furthermore, the use of BCID2 panel allowed us to have rapid identification of resistance genes in period 2, highlighting the rise of CPE. This period coincides with the increase of bacterial resistance during the COVID-19 pandemic [32] and highlights the importance of adjusting the empiric approach for antimicrobial treatment [14,33]. Empirical therapy protocols in our center are based on the initial use of piperacillin/tazobactam or meropenem in a context of high ESBL prevalence. However, these changes in the epidemiology of BSI episodes make it necessary to collect more data by center and country within the region in order to update the mentioned protocols [4].

The overall mortality in patients with episodes of bacteremia due to CPE was 100%, similar to the report by Jaiswal et al. in India [34]. Inadequate empirical antimicrobial therapy in high-risk neutropenic patients with gram-negative bacteremia has been reported to be associated with increased mortality [35,36]. We observed high rates of appropriateness in empirical therapy according to the initial consensus algorithm on the two intervention periods, but appropriateness de-escalation or continuation decreased in period 2 since addressing emerging resistance mechanisms were not contemplated. Additionally, three cases of relapse of CPE bacteremia were documented in patients with colistin-based regimens. Therefore, it is necessary to evaluate the real effectiveness of colistin in these scenarios.

The targeted treatment of infections caused by CPE on the hematology ward was difficult because our center does not count with alternative drugs to colistin or tigecycline, which are recommended by some guidelines [33,37]. For example, ceftazidime/avibactam is recommended as an alternative for severe infections due to KPC-producing or OXA-48 producing *Enterobacteriaceae* for the management of FN in patients with hematological malignancies, despite the lack of well-designed comparative studies [33,38]. The ATLAS global surveillance program from 2017 to 2019 collected information from the Latin American region and reported in vitro susceptibility to ceftazidime/avibactam and colistin in 99.4% and 74.9% in non-metallo-ẞ-lactamase CPE (n = 358), respectively [39]. In a scenario like Peru, where there is also an emergence of colistin-resistant *Klebsiella pneumoniae* strains [40], it is necessary to consider the inclusion of new ẞ-lactam-ẞ-Lactamase Inhibitor combinations or novel agents (e.g., cefiderocol), considering the increase in bone marrow transplant patients (as seen in period 2) [41]. Based on the emergence of CPE and the increase in hematopoietic transplant recipients in our hospital, it is necessary to reinforce the active surveillance system for multidrug-resistant organisms (MDROs) (pre-transplant and pre-engraftment), and through longitudinal studies, the impact of new combinations of antimicrobials administered early in colonized patients could be evaluated.

Almenara Hospital has had an operating ASP since 2017, with initial objectives being met after its implementation, such as the reduction in the antimicrobial consumption rates of imipenem and vancomycin [20]. These antibiotics exert selection pressure for the appearance of MDROs. In intervention period 2, no episodes of MDR or XDR *Pseudomonas aeruginosa* bacteremia were detected. This could be a trend associated with the effect of the ASP intervention. Medium-term objectives such as changes in the resistance profile of some germs such as *Pseudomonas aeruginosa* were documented in other areas of our center (ICU) in 2018 [42]. In light of the emergence of multidrug-resistant *Enterobacteriaceae* and evolving resistance patterns of *Pseudomonas aeruginosa* observed in our hospital, there is a need to explore non-carbapenem options for treating this group of pathogens. Ceftolozane/tazobactam has been recommended by some guidelines as targeted therapy against Difficult-To-Treat Resistance (DTR) *Pseudomonas aeruginosa*. However, further prospective evaluation is necessary to determine its efficacy in managing febrile neutropenia [33,38].

These data confirm the enormous gap in conventional microbiological diagnosis in our institution and a molecular microbiological approach, for which we consider that resuming efforts should be prioritized to continue with ASP strategies. The hematology ward was one of the first to join the continuous effort of the strategy through the development of algorithms for the empirical treatment of FN, the discussion of antifungal use strategies, dosing of vancomycin trough levels, among other interventions to the group of patients with hematological malignancies (including autologous transplants).

**Figure 2:**
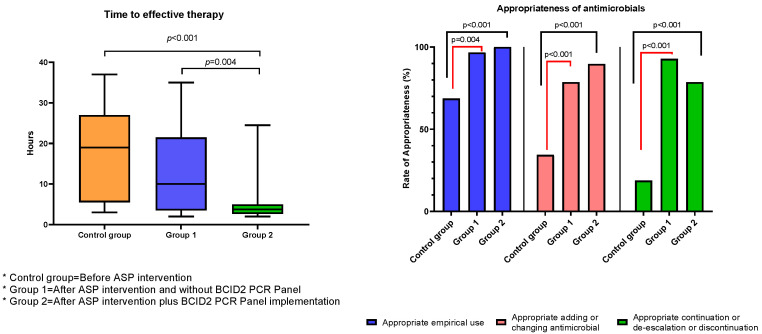
Time to effective therapy and appropriateness of antimicrobials. Comparison between intervention periods and control group. Abbreviations: ASP = antimicrobial stewardship program, BCID2 = Blood Culture Identification 2 (BCID2) Panel.

Our study has some limitations. First, it is likely that patients in the intervention period may have more isolates or different epidemiology based on previous ASP interventions, which reinforced microbiological aspects (timely collection of blood cultures, sample quality, among others) and the prescription of empirical antimicrobials based on a treatment algorithm. This could influence the better selection and de-escalation of antimicrobials; however, this does not translate into a lower selection of MDROs. Additionally, this is a single-center study, with a small sample size and, in period 2, is limited by the budget allocated to the study. The results and outcomes should be interpreted with caution. However, to reduce selection bias, we were rigorous in identifying cases in the control and period 1. Nevertheless, there are potential unmeasured confounders such as limitations in hospitalizations due to outbreaks (the service only has 11 beds), variations in hospital stay periods, and the contamination ratio of blood cultures, among others. Second, many of the ASP strategies, as well as in other local institutions and worldwide, have been affected during the COVID-19 pandemic. ASP members were distributed by contingency issues. Additionally there is a decreased laboratory capacity due to staff desertion or redistribution of functions [43]. Third, it was not possible to determine if the decrease in indications for antibacterial prophylaxis with quinolones contributed to CPE increase in the intervention period 2. Although there are reviews that indicate that their use has no impact on the survival of patients with FN, it does not influence the selection pressure by MDROs [44], for which it is necessary to re-evaluate alternative strategies in contexts of the high prevalence of ESBL. Fourth, despite the local initiatives of our hospital in order to consolidate an ASP, there are still no regulations for all the social security centers in Peru and reports of clinical or impact indicators of these programs are still very scarce. We believe that political barriers and the availability of human resources assigned exclusively to these programs limit interventions. Nevertheless, our study possesses several strengths. It is the first study to describe the impact of an ASP intervention over a 5-year period in Peru, demonstrating an increase in the appropriateness rate of antimicrobial use at various time points when compared to the control period. Additionally, the BCID2 intervention period significantly shortened the time to effective therapy (Figure 2). Furthermore, the initial reduction in hospital LOS observed in our study highlights the potential of ASP to reduce healthcare costs and lays the foundation for future interventions aimed at strengthening these programs. Through further research in this area, we can enhance our understanding of the impact of ASP on hospital LOS and improve patient outcomes.

## 4. Materials and Methods

Study design. This was a pre-post-quasi experimental single-center study conducted at the Guillermo Almenara Irigoyen National Hospital in Lima, Peru. This is a reference center for the insured population in Peru with a capacity of 1116 beds and 23,113 annual discharges. The study was conducted according to the guidelines of the Declaration of Helsinki, and approved by the Institutional Review Board of Guillermo Almenara Irigoyen National Hospital (protocol code 27-2021).

The study population included all patients older than 18 years old who were admitted to the adult hematology ward (11 beds), with a clinical-laboratory diagnosis of FN and bloodstream infection (BSI) from October 2015 to October 2022. Patients with identification of bacteremia prior to admission to the adult hematology ward, previous bacteremia by the same microorganism, and isolates that were considered contaminants by the ASP team were excluded.

The following three groups were considered: patients with BSI from October 2015 to October 2018 (before ASP intervention—control group), patients with BSI from October 2018 to October 2021 (after ASP intervention and without BCID2 PCR Panel—group 1), and patients with BSI from October 2021 to October 2022 (after ASP intervention plus BCID2 PCR Panel implementation—group 2). Convenience sampling was performed in group 2 for the budget designated for this study. All consecutive cases that met the inclusion criteria for period 2 were considered.

Procedures. Evaluation of patients for inclusion in the study was performed by consulting the database of positive blood cultures from the microbiology laboratory. Only the first positive blood culture was considered for each patient. Once the patients were identified, the electronic hospital record or clinical history was used to collect the variables of interest. Patients with incomplete records of clinical, microbiological, or treatment outcomes were not considered.

Data in the intervention period (period 2) were obtained upon discharge of the patient and the information obtained was compiled in a database stored in the Excel v.2019 program. To obtain the total number of isolates in period 2, the isolates obtained by both BCID2 and the microbiology laboratory were combined. Informed consent was not used, since the interventions for the microbiological diagnostic approach of FN are part of the standard protocols in the institution.

Definitions: Febrile neutropenia (FN) is defined as an oral temperature of >38.3 °C or two consecutive readings of >38.0 °C for 2 h and an absolute neutrophil count (ANC) of <0.5 × 10^9^/L, or they were expected to fall below 0.5 × 10^9^/L [1].

Bloodstream infection (BSI), demonstrated by positive blood culture, included primary bacteremia/fungemia and secondary bacteremia/fungemia observed while they were neutropenic in the same episode of FN. Contamination is considered to be the isolation of common species in a single blood culture bottle (e.g., coagulase-negative *Staphylococcus*).

The Multinational Association of Supportive Care in Cancer (MASCC) risk index score was applied to determine the risk of serious complications during FN [45]; episodes were classified as high risk if the score was <21 points and as low risk if the score was ≥21 points.

The appropriateness of antimicrobial use was assessed in three steps: 1. Empirical antimicrobial therapy. 2. Addition or change of antimicrobials. 3. Appropriate continuation, de-escalation, or discontinuation.

Intervention strategy: For ASP implementation in 2018, a focus group methodology was used, which included professionals from the infectious disease (ID) unit, microbiology department, infection preventionist, hematologists, clinical pharmacists, and nurses. Based on prioritized axes, functions and strategies by health professional groups were established. The antimicrobial management protocols for FN episodes were revised and agreed upon, as well as antimicrobial and antifungal prophylaxis (infectious disease (ID) unit and hematologists). An ID member of the ASP evaluated the appropriateness of the antimicrobial prescriptions (the rate of appropriateness in starting, adding, switching, de-escalating, and discontinuing antimicrobials was analyzed). Infection preventionist measured compliance with transmission-based isolation precautions, handwashing behavior, and coordinated educational activities to optimize cleaning and disinfection measures. The surveillance of MDR organisms and telephone reporting of results during the intervention period (group 2) were the responsibility of the microbiology staff. Clinical pharmacist developed antimicrobial infusion protocols and the antimicrobial consumption rates. Nurses optimized infusion practices and received training for blood culture collection.

The ASP strategy was both prospective and restrictive antimicrobial audit (for high-cost antimicrobials), however the positive culture review strategy was applied in intervention group 2. Recommendations for changing antimicrobial therapy, if necessary, could be made from 08:00 a.m. to 06:00 p.m., Monday–Friday. Results outside these hours could be performed the next business day. During the intervention period with BCID2, the microbiology laboratory staff communicated the results electronically (WhatsApp telephone network) to the ASP team. The results were published in the medical records after verbal notification.

Identification and susceptibility testing were performed using conventional phenotypic methods and a VITEK-2 system. The FilmArray panel was granted a trial period at the hospital during 2020–2022. Additional molecular diagnostic methods were carried out in the molecular biology laboratory of the Universidad Peruana de Ciencias Aplicadas (UPC) if necessary. At the time of BCID2 implementation, the presence of carbapenemases was detected using the modified carbapenem inactivation method (mCIM), and the immunochromatographic test (K-set CORIS Bio-Concept RESIST-4 O.K.N.V). The FilmArray BCDI2 panel (BioFire Diagnostics, Salt Lake City, UT, USA.) was processed from positive blood cultures as of 23 October 2021. The panel can identify within the gram-negative bacteria *Acinetobacter calcoaceticus baumannii* complex, *Bacteroides fragilis*, Enterobacteriaceae: *Enterobacter cloacae* complex, *Escherichia coli*, *Klebsiella aerogenes*, *Klebsiella oxytoca*, *Klebsiella pneumoniae* group, *Proteus*, *Salmonella*, *Serratia marcescens*, *Haemophilus influenzae*, *Neisseria meningitidis*, *Pseudomonas aeruginosa*, *Stenotrophomonas maltophilia*; within gram positives to: *Enterococcus faecalis*, *Enterococcus faecium*, *Listeria monocytogenes*, *Staphylococcus*: *Staphylococcus aureus*, *Staphylococcus epidermidis*, *Staphylococcus lugdunensis*, *Streptococcus: Streptococcus agalactiae*, *Streptococcus pneumoniae*, *Streptococcus pyogenes*, and yeasts such as *Candida albicans*, *Candida auris*, *Candida glabrata*, *Candida krusei*, *Candida parapsilosis*, *Candida tropicalis*, and *Cryptococcus neoformans/gattii*. It can also detect the resistance genes: IMP, KPC, OXA-48, NDM, VIM, mcr-1, CTX-M, mecA/C, MREJ (MRSA), and vanA/B. Results were available within 1 h of processing.

The discrepancies between BCID2 and conventional culture and phenotypic antimicrobial susceptibility test results were described. Additionally, the resistance profiles of the BSI episodes were compared between the three periods.

Outcomes. The primary outcome was the comparison of times to effective therapy between the three groups. The time to effective therapy was defined as the period between the index blood culture collection and the receipt of the initial dose of an antimicrobial shown to exhibit activity against the patient-specific organism based on in vitro susceptibility results, with intermediate results considered ineffective [24].

Clinical endpoints were compared between groups and included in-hospital mortality (all cause), 30-day all-cause readmission, hospital length of stay (LOS), and LOS following first positive blood culture. All-cause mortality was defined as death resulting from any cause at the end of hospitalization. A relapse of BSI was defined as the reoccurrence of the same organism in a blood culture within 30 days after the end of treatment.

Statistical analysis. Statistical comparisons were made between intervention groups and the control group using the Kruskal–Wallis or the Mann–Whitney test for continuous variables, as appropriate. The chi2 test or Fisher’s exact test was obtained for dichotomous variables. Statistical analysis was performed using Stata SE 15.0 software for Windows (College Station, TX, USA). Graphs were created with GraphPad Prism 9.0.0. A *p* value ≤ 0.05 was considered statistically significant.

## 5. Conclusions

In addition to the lack of local studies documenting the microbiological profile of FN episodes in Peru, we considered that adding syndromic panels-based testing will allow for the consolidation of ASP strategies. Likewise, this study could generate evidence for the subsequent implementation of these tests on an institutional level.

## Figures and Tables

**Table 1:** Demographics and baseline characteristics of patients with bloodstream infections.

Characteristic		Before ASP Intervention *n* = 32	After ASP Intervention *n* = 30	After ASP Intervention Plus BCID2 *n* = 31	*p* Value
Sex					
	Male	20 (62.5%)	20 (66.7%)	10 (32.3%)	0.013
Age, mean (IQR)		46.7 (38–57.5)	45.3 (37–56)	50 (40–62)	0.279
Comorbidities					
	Diabetes mellitus	2 (6.3%)	1 (3.3%)	1 (3.2%)	1.000
	Liver disease	2 (6.3%)	0	2 (6.5%)	0.541
	Renal replacement therapy	1 (3.1%)	0	3 (9.7%)	0.216
	HIV/AIDS	0	1 (3.3%)	0	0.323
	Cerebrovascular disease	1 (3.1%)	1 (3.3%)	1 (3.2%)	1.000
	Peripheral vascular disease	0	2 (6.7%)	0	0.102
	Chronic heart failure	1 (3.1%)	0	0	1.000
	Hypertension	1 (3.1%)	2 (6.7%)	4 (12.9%)	0.341
	Connective tissue disease	0	2 (6.7%)	0	0.102
Hematological malignancy					
	Acute myeloid leukemia	13 (40.6%)	12 (40%)	16 (51.6%)	0.585
	Acute lymphocytic leukemia	13 (40.6%)	14 (46.7%)	10 (32.2%)	0.513
	Non-Hodgkin’s lymphoma	3 (9.4%)	4 (13.3%)	1 (3.2%)	0.386
	Aplastic anemia	1 (3.1%)	0	2 (6.5%)	0.652
	Autologous stem cell transplant	0	0	2 (6.5%)	0.21
	Multiple myeloma	2 (6.3%)	0	0	0.326
Suspected source of infection					
	Urine	3 (9.4%)	4 (13.3%)	1 (3.2%)	0.386
	Catheter related	11 (34.4%)	6 (20%)	5 (16.1%)	0.199
	Respiratory	6 (18.8%)	5 (16.7%)	5 (16.1%)	0.958
	Intra-abdominal	5 (15.6%)	4 (13.3%)	6 (19.4%)	0.811
	Skin and soft tissue	2 (6.2%)	2 (6.7%)	9 (29.1%)	0.021
	Others	0	0	1 (3.2)	0.656
	Unidentified	5 (15.6%)	9 (30%)	4 (12.9%)	0.193
MASCC score < 21 (high risk)		23 (71.9%)	25 (83.3%)	28 (90.3%)	0.161
Charlson Comorbidity Index, mean (IQR)		2.8 (2–3.5)	3.1 (2–4)	3.5 (3–4)	0.02
Pitt bacteremia score (PBS), mean (IQR)		1.5 (0–2)	1.8 (1–2)	1.8 (0–3)	0.693
Mechanic ventilation		4 (12.5%)	3 (10%)	6 (19.4%)	0.569
Admission to ICU within 48 h of the episode of BSI		3 (9.4%)	2 (6.7%)	2 (6.5%)	1.000
Polymicrobial infection		8 (25%)	7 (23.3%)	8 (25.8%)	0.974
Antibacterial prophylaxis		20 (62.5%)	4 (13.3%)	9 (29.1%)	<0.001
Antifungal prophylaxis		10 (31.3%)	13 (43.3%)	19 (61.3%)	0.055

Abbreviations: ASP = antimicrobial stewardship program, BCID2 = Blood Culture Identification 2 (BCID2) Panel, HIV/AIDS = human immunodeficiency virus/acquired immunodeficiency syndrome, ICU = intensive care unit, IQR = Interquartile Range, MASCC = Multinational Association for Supportive Care in Cancer, BSI = bloodstream infection.

**Table 2:** A description of blood culture pathogens and microbiology-related outcomes.

Parameter	Before ASP Intervention	After ASP Intervention	After ASP Intervention Plus BCID2	*p* Value
Microbiology-related outcomes				
Time to blood culture positivity, h, median (IQR)	18 (15.5–20)	18 (15–21)	11.14 (9.4–15.7) ^b,c^	<0.001
Time to organism identification, h, median (IQR)	55 (50–58)	48 (43–56) ^b^	19.5 (17.8–25.2) ^b,c^	<0.001
Time to in vitro susceptibility results, h, median (IQR)	64 (58–67.5)	62 (56–68)	59.1 (56–64.6)	0.317
Blood culture pathogens ^a^				
Gram-positive bacteria (n = 27)	11/41 (26.8%)	5/37 (13.5%)	11/45 (24.5%)	0.321
*Staphylococcus* spp. (coagulase negative)	5	4	8	
*Staphylococcus aureus*	3	1	0	
*Enterococcus faecium*	2	0	1	
*Streptococcus* spp.	1	0	2	
Gram-negative bacteria (n = 93)	28/41 (68.3%)	32/37 (86.5%)	33/45 (73.3%)	0.158
*E. coli*	8	10	10	
*Klebsiella pneumoniae*	6	5	10	
*Pseudomonas aeruginosa*	7	7	5	
*Acinetobacter baumannii*	2	1	1	
*Citrobacter freundii*	1	0	0	
*Enterobacter cloacae*	1	2	0	
*Acinetobacter lwoffii*	1	3	1	
*Pseudomonas putida*	1	0	1	
*Aeromonas hydrophila*	0	1	0	
*Serratia marcescens*	0	1	2	
*Klebsiella oxytoca*	1	0	2	
*Leminorella richardii*	0	1	0	
*Stenotrophomonas maltophilia*	0	1	0	
*Proteus* spp.	0	0	1	
*Candida* spp. (n = 3)	2/41 (4.9%)	0/0 (0%)	1/45 (2.2%)	0.64
*Candida albicans*	1	0	0	
*Candida tropicalis*	1	0	0	
*Candida kefyr*	0	0	1	

Abbreviations: h = hours, ASP = antimicrobial stewardship program, BCID2 = Blood Culture Identification 2 (BCID2) Panel, IQR = Interquartile Range. ^a^ Data are presented as number of isolates/total of isolates by period (percent). ^b^ Statistically significant compared to first period: before ASP intervention ^c^ Statistically significant between the two intervention groups. The presented *p*-value resulted from the comparison between the three groups.

**Table 3:** Discrepancies between BCID2 and conventional culture and phenotypic antimicrobial susceptibility test results.

Patient	BCID2 Result	Conventional Culture and Phenotypic Susceptibility Testing Result(s)
Polymicrobial cultures		
Patient #3	*Klebsiella pneumoniae*, *Acinetobacter calcoaceticus-baumannii* complex, *E. coli*, *Streptococcus* spp.	*Klebsiella pneumoniae*
Patient #11	*Staphylococcus epidermidis* (gen mec A/C), *Streptococcus* spp.	Methicillin-resistant *Staphylococcus epidermidis*
Patient #31	*E. coli*/*Klebsiella pneumoniae* (CTX-M), *Proteus* spp., *Serratia marcescens*	ESBL-producing *E. coli*, *Serratia marcescens*
Discrepancies between resistance genes identified by BCID2 panel and phenotypic antimicrobial susceptibility test results		
Patient #4	*Klebsiella oxytoca*	ESBL-producing *Klebsiella oxytoca*
Patient # 25	*Staphylococcus epidermidis*	Methicillin-resistant *Staphylococcus epidermidis*
Discrepancy in organism identification		
Patient #10	Non-identified	*Acinetobacter lwoffii*
Patient #15	Non-identified	*Pseudomonas putida*
Patient #27	*Staphylococcus epidermidis* (gen mec A/C)	*Candida kefyr*

Abbreviations: BCID2= Blood Culture Identification 2 (BCID2) Panel, ESBL= Extended-spectrum beta-lactamase.

**Table 4:** Antibiotic resistance profiles of bacterial and fungal bloodstream infections.

Parameter		Before ASP Intervention	After ASP Intervention	After ASP Intervention Plus BCID2
Blood culture pathogens				
*Staphylococcus* spp. (coagulase negative) meticillin-resistance				
		4/4 (100%)	3/4 (75%)	7/8 (87.5%) *
*Staphylococcus aureus* meticillin-resistance				
		3/3 (100%)	1/1 (100%)	0
*Enterobacteriaceae* ESBL CPE				
		8/17 (47.1%)	8/19 (42.1%)	9/25 (36%) **
		0	0	5/25 (20%) ***
*Pseudomonas aeruginosa* MDR XDR DTR Susceptible				
		3/7 (42.9%)	0	0
		4/7 (57.1%)	2/7 (28.6%)	0
		0	1/7 (14.3%)	0
		0	4/7 (57.1%)	5/5 (100%)
*Acinetobacter baumannii* MDR XDR Susceptible				
		1/2 (50%)	0	0
		0	0	0
		1/2 (50%)	1/1 (100%)	1/1(100%)
*Enterococcus faecium* Vancomycin resistant				
		2/2 (100%)	0	1/1 (100%) ****
*Candida* Resistant to azoles				
		1/2 (50%)	0	1/1 (100%)

Abbreviations: ASP = antimicrobial stewardship program, BCID2 = Blood Culture Identification 2 (BCID2) Panel, ESBL = Extended-spectrum beta-lactamase, CPE = Carbapenemase-producing Enterobacteriaceae, MDR = Multi-Drug Resistance, XDR = Extensive Drug Resistance, DTR = Difficult-to-Treat Resistance. * Detection of gen mec A/C in six isolates. ** Detection of gen CTX-M in eight isolates. *** Detection of gen KPC in four isolates, and OXA-48 in one isolate. **** Resistant to linezolid and the detection of gen van A/van B.

**Table 5:** The appropriateness of antimicrobials.

	Before ASP Intervention	After ASP Intervention	After ASP Intervention Plus BCID2	*p* Value
Appropriateness of antimicrobials ^a^				
Appropriate empirical use	22/32 (68.7%)	29/30 (96.7%) ^b^	31/31 (100%) ^b^	<0.001
Appropriate adding or changing antimicrobial	11/32 (34.4%)	22/28 (78.6%) ^b^	26/29 (89.7%) ^b^	<0.001
Appropriate continuation or de-escalation or discontinuation	6/32 (18.8%)	26/28 (92.8%) ^b^	22/28 (78.6%) ^b^	<0.001

Abbreviations: ASP = antimicrobial stewardship program, BCID2 = Blood Culture Identification 2 (BCID2) Panel. ^a^ Data are presented as number (percent) of patients. ^b^ Statistically significant compared to first period: before ASP intervention. The presented *p*-value resulted from the comparison between the three groups.

**Table 6:** Treatment and clinical outcomes.

Parameter	Before ASP Intervention *n* = 32	After ASP Intervention *n*= 30	After ASP Intervention Plus BCID2 *n*= 31	*p* Value
Treatment-related outcomes				
Time to effective therapy, h, median (IQR) (*n* = 78)	19 (6–27)	10 (4–20) ^b^	3.75 (2.75–5) ^b,c^	<0.001
Relapse of bacteremia ^a^	5 (15.6%)	0	5 (16.1%)	0.051
Clinical outcomes				
Hospital length of stay (LOS), number of days, median (IQR)	33 (27–50)	27 (23–37)	28 (23–32) ^b^	0.042
LOS, number of days, following first positive blood culture, median (IQR)	10 (6.5–12)	11 (6–19)	11 (8–18)	0.281
In-hospital mortality (all cause)^a^	9 (28.1%)	5 (16.7%)	11 (35.5%)	0.248
30-day all-cause hospital readmission (*n* = 68) ^a^	12 (52.1%)	9 (36%)	7 (35%)	0.419

Abbreviations: h = hours, ASP = antimicrobial stewardship program, BCID2 = Blood Culture Identification 2 (BCID2) Panel, IQR = Interquartile Range. ^a^ Data are presented as number (percent) of patient, unless specified otherwise. ^b^ Statistically significant compared to first period: before ASP intervention. ^c^ Statistically significant between the two intervention groups. The presented *p*-value resulted from the comparison between the three groups.

## Data Availability

The data supporting the reported results are available from the corresponding author upon reasonable request.

## References

[1] Klastersky J., de Naurois J., Rolston K., Rapoport B., Maschmeyer G., Aapro M., Herrstedt J., on behalf of the ESMO Guidelines Committee (2016). Management of febrile neutropaenia: ESMO Clinical Practice Guidelines. Ann. Oncol..

[2] Carvalho A.S., Lagana D., Catford J., Shaw D., Bak N. (2020). Bloodstream infections in neutropenic patients with haematological malignancies. Infect. Dis. Health.

[3] Murray C.J., Ikuta K.S., Sharara F., Swetschinski L., Aguilar G.R., Gray A., Han C., Bisignano C., Rao P., Wool E. (2022). Global burden of bacterial antimicrobial resistance in 2019: A systematic analysis. Lancet.

[4] Lalaoui R., Javelle E., Bakour S., Ubeda C., Rolain J.-M. (2020). Infections Due to Carbapenem-Resistant Bacteria in Patients With Hematologic Malignancies. Front. Microbiol..

[5] Nesher L., Rolston K.V.I. (2014). The current spectrum of infection in cancer patients with chemotherapy related neutropenia. Infection.

[6] Trecarichi E.M., Pagano L., Candoni A., Pastore D., Cattaneo C., Fanci R., Nosari A., Caira M., Spadea A., Busca A. (2015). Current epidemiology and antimicrobial resistance data for bacterial bloodstream infections in patients with hematologic malignancies: An Italian multicentre prospective survey. Clin. Microbiol. Infect..

[7] Wang L., Wang Y., Fan X., Tang W., Hu J. (2015). Prevalence of Resistant Gram-Negative Bacilli in Bloodstream Infection in Febrile Neutropenia Patients Undergoing Hematopoietic Stem Cell Transplantation. Medicine.

[8] Kara Ö., Zarakolu P., Aşçioğlu S., Etgül S., Uz B., Büyükaşik Y., Akova M. (2015). Epidemiology and emerging resistance in bacterial bloodstream infections in patients with hematologic malignancies. Infect. Dis..

[9] El-Mahallawy H., Samir I., Fattah R.A., Kadry D., El Kholy A. (2014). Source, pattern and antibiotic resistance of blood stream infections in hematopoietic stem cell transplant recipients. J. Egypt. Natl. Cancer Inst..

[10] Garzón J.R., Isaza N., Posada A., Mendez R., Arenas J., Ardila M.P., Cárdenas A., Barrera V., Moreno P., Córdoba I. (2019). Clinical and microbiological characteristics of patients with febrile neutropenia in one Colombian Universitary Hospital. Infectio.

[11] Klastersky J., Ameye L., Maertens J., Georgala A., Muanza F., Aoun M., Ferrant A., Rapoport B., Rolston K., Paesmans M. (2007). Bacteraemia in febrile neutropenic cancer patients. Int. J. Antimicrob. Agents.

[12] Righi E., Peri A.M., Harris P.N.A., Wailan A.M., Liborio M., Lane S.W., Paterson D.L. (2017). Global prevalence of carbapenem resistance in neutropenic patients and association with mortality and carbapenem use: Systematic review and meta-analysis. J. Antimicrob. Chemother..

[13] Hinojosa-Andía L.J., del Carpio-Jayo D. (2014). Bacteremia associated with febrile neutropenia in hematology-oncology patients, bacterial spectrum and antibiotic susceptibility pattern. Rev. Med. Hered..

[14] Nouér S.A., Nucci M., Anaissie E. (2015). Tackling antibiotic resistance in febrile neutropenia: Current challenges with and recommendations for managing infections with resistant Gram-negative organisms. Expert Rev. Hematol..

[15] Wright J.D., Neugut A.I., Ananth C.V., Lewin S.N., Wilde E.T., Lu Y.-S., Herzog T.J., Hershman D.L. (2013). Deviations From Guideline-Based Therapy for Febrile Neutropenia in Cancer Patients and Their Effect on Outcomes. JAMA Intern. Med..

[16] Baugh C.W., Wang T.J., Caterino J.M., Baker O.N., Brooks G.A., Reust A.C., Pallin D.J. (2017). Emergency Department Management of Patients with Febrile Neutropenia: Guideline Concordant or Overly Aggressive?. Acad. Emerg. Med..

[17] O’Horo J.C., Marcelin J., Abu Saleh O.M., Barwise A.K., Odean P.M., Rivera C., Tande A.J., Wilson J.W., Osmon D.R., Tosh P.K. (2019). Standardizing Febrile Neutropenia Management: Antimicrobial Stewardship in the Hematologic Malignancy Population. J. Oncol. Pract..

[18] Rosa R.G., Goldani L.Z., Dos Santos R.P. (2014). Association between adherence to an antimicrobial stewardship program and mortality among hospitalised cancer patients with febrile neutropaenia: A prospective cohort study. BMC Infect. Dis..

[19] Madran B., Keske Ş., Tokça G., Dönmez E., Ferhanoğlu B., Çetiner M., Mandel N.M., Ergönül Ö. (2018). Implementation of an antimicrobial stewardship program for patients with febrile neutropenia. Am. J. Infect. Control.

[20] Hernández-Gómez C., Hercilla L., Mendo F., Pérez-Lazo G., Contreras E., Ramírez E., Flores W., Julca Á., Chuquiray N., Arenas B. (2019). Antimicrobial Stewardship programs in Peru: A fundamental agreement. Rev. Chil. Infectología.

[21] CDC (2019). Core Elements of Hospital Antibiotic Stewardship Programs.

[22] Patel R., Fang F.C. (2018). Diagnostic Stewardship: Opportunity for a Laboratory–Infectious Diseases Partnership. Clin. Infect. Dis..

[23] Peri A.M., Ling W., Furuya-Kanamori L., Harris P.N.A., Paterson D.L. (2022). Performance of BioFire Blood Culture Identification 2 Panel (BCID2) for the detection of bloodstream pathogens and their associated resistance markers: A systematic review and meta-analysis of diagnostic test accuracy studies. BMC Infect. Dis..

[24] MacVane S.H., Nolte F.S. (2016). Benefits of Adding a Rapid PCR-Based Blood Culture Identification Panel to an Established Antimicrobial Stewardship Program. J. Clin. Microbiol..

[25] Egli A., Osthoff M., Goldenberger D., Halter J., Schaub S., Steiger J., Weisser M., Frei R. (2015). Matrix-assisted laser desorption/ionization time-of-flight mass spectrometry (MALDI-TOF) directly from positive blood culture flasks allows rapid identification of bloodstream infections in immunosuppressed hosts. Transpl. Infect. Dis..

[26] Szymankiewicz M., Nakonowska B. (2018). Rapid Detection of Bloodstream Pathogens in Oncologic Patients with a FilmArray Multiplex PCR Assay: A Comparison with Culture Methods. Pol. J. Microbiol..

[27] Buss B.A., Baures T.J., Yoo M., Hanson K.E., Alexander D.P., Benefield R.J., Spivak E.S. (2018). Impact of a Multiplex PCR Assay for Bloodstream Infections with and without Antimicrobial Stewardship Intervention at a Cancer Hospital. Open Forum Infect. Dis..

[28] Rosa R., Suarez J.F., Bravo G., A Morillas-Rodriguez J., Anderson A.D., Camargo J.F., Abbo L. (2019). Challenges in Antimicrobial Stewardship: Rapid Diagnostics and Optimization of Therapy Among Immunocompromised Patients. Open Forum Infect. Dis..

[29] Foster R.A., Kuper K., Lu Z.K., Bookstaver P.B., Bland C.M., Mahoney M.V. (2017). Pharmacists’ Familiarity with and Institutional Utilization of Rapid Diagnostic Technologies for Antimicrobial Stewardship. Infect. Control Hosp. Epidemiol..

[30] Nathwani D., Varghese D., Stephens J., Ansari W., Martin S., Charbonneau C. (2019). Value of hospital antimicrobial stewardship programs [ASPs]: A systematic review. Antimicrob. Resist. Infect. Control.

[31] Marcos-Carbajal P., Salvatierra G., Yareta J., Pino J., Vásquez N., Diaz P., Martínez I., Asmat P., Peralta C., Huamani C. (2021). Microbiological and molecular characterization of antimicrobial resistance in uropathogenic Escherichia coli from peruvian public hospitals. Rev. Peru. Med. Exp. Salud Publica.

[32] Al Sulayyim H.J., Ismail R., Al Hamid A., Ghafar N.A. (2022). Antibiotic Resistance during COVID-19: A Systematic Review. Int. J. Environ. Res. Public Health.

[33] Gudiol C., Aguilar-Guisado M., Azanza J.R., Candel F.J., Cantón R., Carratalà J., Garcia-Vidal C., Jarque I., Lizasoain M., Gil-Bermejo J.M. (2020). Executive summary of the consensus document of the Spanish Society of Infectious Diseases and Clinical Microbiology (SEIMC), the Spanish Network for Research in Infectious Diseases (REIPI) and the Spanish Society of Haematology and Haemotherapy (SEHH) on the management of febrile neutropenia in patients with hematological malignancies. Enferm. Infecc. Microbiol. Clínica.

[34] Jaiswal S.R., Gupta S., Kumar R.S., Sherawat A., Rajoreya A., Dash S.K., Bhagwati G., Chakrabarti S. (2018). Gut Colonization with Carbapenem Resistant Enterobacteriaceae Adversely Impacts the Outcome in Patients with Hematological Malignancies: Results of a Prospective Surveillance Study. Mediterr. J. Hematol. Infect. Dis..

[35] Martinez-Nadal G., Puerta-Alcalde P., Gudiol C., Cardozo C., Albasanz-Puig A., Marco F., Laporte-Amargós J., Moreno-García E., Domingo-Doménech E., Chumbita M. (2020). Inappropriate Empirical Antibiotic Treatment in High-risk Neutropenic Patients With Bacteremia in the Era of Multidrug Resistance. Clin. Infect. Dis..

[36] Chumbita M., Puerta-Alcalde P., Yáñez L., Cuesta M.A., Chinea A., Morales I.E., Abellán P.F., Gudiol C., Guerreiro M., González-Sierra P. (2022). Resistance to empirical β-lactams recommended in febrile neutropenia guidelines in Gram-negative bacilli bloodstream infections in Spain: A multicentre study. J. Antimicrob. Chemother..

[37] Satlin M.J., Weissman S.J., Carpenter P.A., Seo S.K., Shelburne S.A. (2021). American Society of Transplantation and Cellular Therapy Series, 1: Enterobacterales Infection Prevention and Management after Hematopoietic Cell Transplantation. Transplant. Cell. Ther..

[38] Clerici D., Oltolini C., Greco R., Ripa M., Giglio F., Mastaglio S., Lorentino F., Pavesi F., Farina F., Liberatore C. (2021). The place of ceftazidime/avibactam and ceftolozane/tazobactam for therapy of haematological patients with febrile neutropenia. Int. J. Antimicrob. Agents.

[39] Karlowsky J.A., Kazmierczak K.M., de Figueiredo Valente M.L.N., Luengas E.L., Baudrit M., Quintana A., Irani P., Stone G.G., Sahm D.F. (2021). In vitro activity of ceftazidime-avibactam against Enterobacterales and Pseudomonas aeruginosa isolates collected in Latin America as part of the ATLAS global surveillance program, 2017–2019. Braz. J. Infect. Dis..

[40] Naomi-Matsuoka A., Vargas M., Ymaña B., Soza G., Pons M.J. (2020). Colistin resistance in multidrug-resistant klebsiella pneumoniae strains at a perinatal maternal institute in Lima, Peru, 2015–2018. Rev. Peru. Med. Exp. Salud Publica.

[41] Sahitya D.S.K., Jandiyal A., Jain A., Senapati J., Nanda S., Aggarwal M., Kumar P., Mohapatra S., Ray P., Malhotra P. (2021). Prevention and management of carbapenem-resistant Enterobacteriaceae in haematopoietic cell transplantation. Ther. Adv. Infect. Dis..

[42] Pérez-Lazo G., Abarca-Salazar S., Lovón R., Rojas R., Ballena-López J., Morales-Moreno A., Flores-Paredes W., Arenas-Ramírez B., Illescas L.R. (2021). Antibiotic Consumption and Its Relationship with Bacterial Resistance Profiles in ESKAPE Pathogens in a Peruvian Hospital. Antibiotics.

[43] Rawson T.M., Moore L.S.P., Castro-Sanchez E., Charani E., Davies F., Satta G., Ellington M.J., Holmes A.H. (2020). COVID-19 and the potential long-term impact on antimicrobial resistance. J. Antimicrob. Chemother..

[44] Mikulska M., Averbuch D., Tissot F., Cordonnier C., Akova M., Calandra T., Ceppi M., Bruzzi P., Viscoli C., Aljurf M. (2018). Fluoroquinolone prophylaxis in haematological cancer patients with neutropenia: ECIL critical appraisal of previous guidelines. J. Infect..

[45] Freifeld A.G., Bow E.J., Sepkowitz K.A., Boeckh M.J., Ito J.I., Mullen C.A., Raad I.I., Rolston K.V., Young J.-A.H., Wingard J.R. (2011). Clinical Practice Guideline for the Use of Antimicrobial Agents in Neutropenic Patients with Cancer: 2010 Update by the Infectious Diseases Society of America. Clin. Infect. Dis..

